# Expression profiles and function analysis of microRNAs in postovulatory aging mouse oocytes

**DOI:** 10.18632/aging.101219

**Published:** 2017-04-08

**Authors:** Tian-Yang Wang, Jie Zhang, Jiang Zhu, Hua-Yu Lian, Hong-Jie Yuan, Min Gao, Ming-Jiu Luo, Jing-He Tan

**Affiliations:** ^1^ College of Animal Science and Veterinary Medicine, Shandong Agricultural University, Tai-an City 271018, P. R. China

**Keywords:** microRNA, oocyte, aging, signaling pathway, molecular function

## Abstract

In this study, microRNA (miRNA) profiles in postovulatory aging mouse oocytes were analyzed by microarray screening and RT-qPCR. Hierarchical cluster analysis on the microarray data and KEGG pathway enrichment analysis on the mRNAs targeted by differentially expressed (DE) miRNAs between two adjacent egg-ages suggest that while only a mild alteration in miRNA expression occurred from 13 to 18 h, a great change took place from 18 to 24 h post hCG injection. Theoretical exploration on functions of the predicted target genes suggest that KEGG pathways enriched by 13-18 h DE miRNAs are correlated with early events of oocyte aging while pathways most enriched by 18-24 h or 24-30 h DE miRNAs are correlated with the late symptoms of aged oocytes. Experimental verification on functions of the key proteins predicted by the KEGG analysis and injection of miR-98 mimics or inhibitors further confirmed that miRNAs played stimulatory/inhibitory roles in postovulatory oocyte aging. In conclusion, marked changes in miRNA expression are associated with significant alterations in function and morphology of postovulatory aging oocytes.

## INTRODUCTION

If not fertilized in time after ovulation, mammalian oocytes undergo a time-dependent process of aging both in vivo and in vitro. The postovulatory oocyte aging has marked detrimental effects on embryo development and offspring [1]. However, the mechanisms for post-ovulatory oocyte aging are not fully understood.

Studies have revealed the presence of microRNAs (miRNAs) in mammalian oocytes during their growth and maturation [2-4]. The presence and spatio-temporal expression of miRNAs and miRNA processing machinery genes in oocytes and preimplantation embryos have evidenced the involvement of miRNAs in growth and maturation of oocytes, early embryo development, stem cell lineage differentiation and implantation [5,6]. However, functional analysis has concluded that miRNAs are ineffective in mouse oocytes and early embryos [7]. Furthermore, recent studies demonstrate that miRNA function is suppressed in mouse oocytes, which suggests that endo-siRNAs, not miRNAs, are essential for female meiosis [8-10]. Thus, whether miRNAs function in mammalian oocytes remains to be clarified.

It is known that postovulatory oocyte aging leads to apoptosis. The expression of the antiapoptotic protein BCL2 was gradually reduced during oocyte aging [11-13]. Injection of sperm cytosolic factor triggered cell death, rather than activation, in aged oocytes. Furthermore, the aged oocytes exhibited extensive cytoplasmic and DNA fragmentation, a prominent decrease in the amounts of Bcl-2 mRNA and protein, and activation of protein caspases [11,14,15]. Since it has been established that miRNAs repress the expression of either pro-apoptotic or antiapoptotic genes to produce antiapoptotic or pro-apoptotic effects, respectively [16], we propose that miRNAs may be involved in oocyte aging.

Changes in protein profiling were observed during postovulatory oocyte aging [17]. Both transcriptional and post-transcriptional regulation can lead to alteration of gene expression. Because transcription is inhibited in mature oocytes, the post-transcriptional regulation may be the primary source for alteration of gene expression in aging oocytes. During oocyte maturation, maternal mRNAs are accumulated in the cytoplasm [18]. Most of these maternal mRNAs are in a masked state, and the translation of these masked mRNAs in mature oocytes is regulated at the post-transcriptional level [19]. Since miRNAs function by causing mRNA translational inhibition or degradation [20,21], it is reasonable to assume that miRNAs may take part in the regulation of maternal mRNA translation in aging oocytes.

The objective of the current study was to provide evidence that miRNAs are involved in postovulatory oocyte aging. To this end, miRNA expression profiles in mouse oocytes aging for different times were first analyzed by microarray screening and RT-qPCR. Hierarchical cluster analysis on the microarray data and KEGG pathway enrichment analysis on the mRNAs targeted by differentially expressed (DE) miRNAs between two adjacent egg-ages were then carried out to explore the function of miRNAs in oocyte aging. Finally, functional verification of key proteins predicted by the KEGG pathway enrichment analysis and injection of miRNA mimics or inhibitors were conducted to confirm the role of miRNAs in oocyte aging. The results suggest that marked changes in miRNA expression are associated with significant alterations in function and morphology of postovulatory aging oocytes.

## RESULTS

### Collection and egg-age verification of in vivo aging oocytes used for miRNA microarray assay

At each time point after hCG injection, 8 superovulated mice were sacrificed and about 240 oocytes were recovered on each experimental day. Around 30 oocytes were randomly taken from the 240 oocytes and subjected to ethanol-alone activation to verify the age of the oocytes. Whereas none of the freshly ovulated (13-h) oocytes was activated, activation rates increased significantly at 18 h (54%) and reached the maximum (97%) at 24 h post hCG injection. About 40% of the oocytes recovered at 30 h after hCG underwent cytoplasmic fragmentation. The results confirmed the age of the oocytes recovered at each time point after hCG injection.

### Microarray assay of miRNA expression profiles in oocytes aging for different times

**Figure 1:**
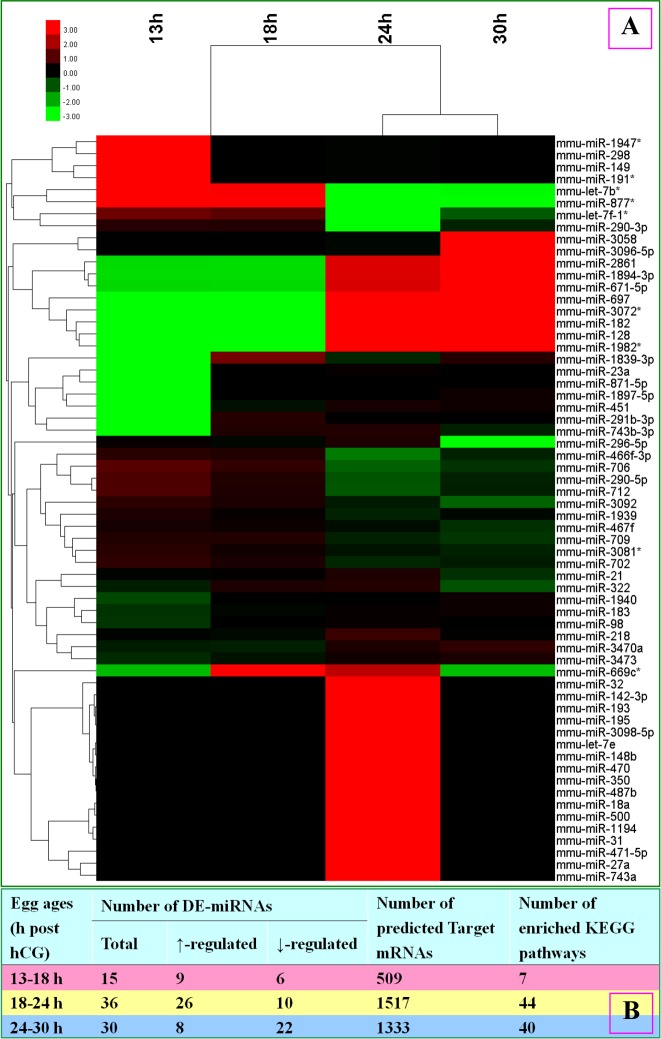
A microarray assay of miRNA expression profiles and a KEGG pathway enrichment analysis of target mRNAs predicted from differentially-expressed (DE) miRNAs in mouse oocytes aging for different times (**A**) Heat map and cluster analysis of miRNA expression in oocytes aging for different times. Red indicates high relative expression and green indicates low relative expression. Fold changes greater than 2 (FC>2) were used as threshold for miRNA differential expression. (**B**) A table shows number of DE miRNAs, number of target mRNAs predicted from DE miRNAs and number of enriched KEGG pathways in oocytes aging for different times.

To determine miRNA expression profiles, oocytes recovered at different times after hCG injection were subjected to a miRNA microarray assay. Briefly, 117, 121, 142 and 127 miRNAs were detected in oocytes collected at 13, 18, 24 and 30 h after hCG injection, respectively. Fold changes greater than 2 (FC>2) were used as threshold for miRNA differential expression. Under the criteria, 62 miRNAs were differentially expressed between at least two egg ages (Fig. 1A). Hierarchical cluster analysis of these 62 miRNAs showed that the 13-h and 18-h oocytes were clustered with a Rescaled Distance Cluster Combine (RDCC) of 1, the 24-h and 30-h oocytes were clustered with a RDCC of 15, and these 2 clusters were further clustered with a RDCC of 25. Specifically, 15 miRNAs were differentially expressed between 13-h and 18-h oocytes (Fig. 1B), among which, 9 were up and 6 were down regulated from 13 to 18 h; 36 miRNAs were differentially expressed between 18-h and 24-h oocytes, of which, 26 increased and 10 decreased; 30 miRNAs were differentially expressed between 24-h and 30-h oocytes, among which, 8 were up and 22 were down regulated. The results suggest that while only a mild alteration in miRNA expression occurred from 13 to 18 h, a great change took place from 18 to 24 h post hCG injection. The number of down-regulated miRNAs increased significantly with increasing post-hCG time suggesting miRNA degradation with oocyte aging.

### RT-qPCR validation of the microarray results

**Figure 2:**
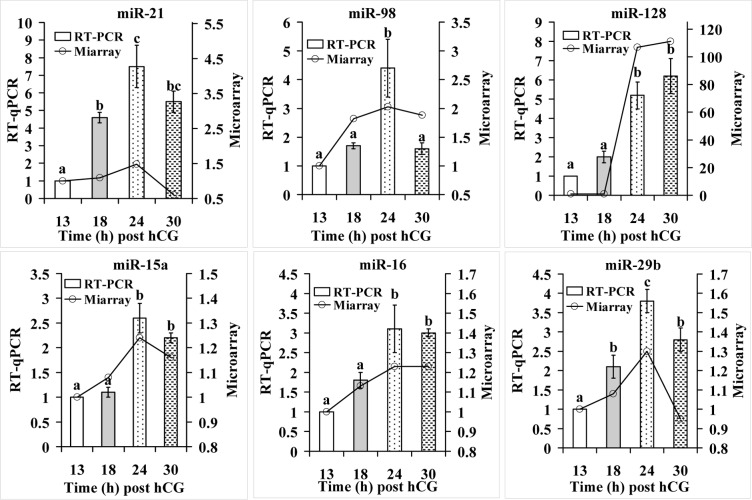
RT-qPCR verification of microarray assay results for apoptosis-related miRNAs Each treatment was repeated 3-4 times and each replicate contained ∼600 oocytes. a-c: Values with a different letter above bars differ significantly (P < 0.05).

From the miRNAs detected by microarray assay, 6 apoptosis-related miRNAs were selected for RT-qPCR assay. Among them, whereas miR-21, miR-98 and miR-128 showed a fold change greater than 2 (FC>2), miR-15a, miR-16 and miR-29b had a fold change less than 2 (FC<2). For all the 6 miRNAs examined, RT-qPCR revealed an expression pattern similar to that detected by the microarray assay (Fig. 2). However, although miR-15a, miR-16 and miR-29b showed a fold change less than 2 during microarray assay, RT-qPCR detected significant alterations between egg-ages. In addition, the alteration amplitude of miR-128 detected by microarray was markedly higher than that detected by RT-qPCR. This might have occurred because miR-128 in 13-h oocytes was undetectable by microarray assay producing a relative level of 0, but it was detected by RT-qPCR with its level set as 1 for control. In all, the results suggest that RT-qPCR results were generally consistent with microarray data, but microarray was less sensitive than RT-qPCR in detecting miRNAs with a low expression.

### Bioinformatical analysis of microarray data

To carry out bioinformatical analysis, a target mRNA prediction was performed using DE miRNAs from oocytes between two adjacent egg-ages. Predicted target mRNAs were then subject to KEGG pathway enrichment analysis. Together, 509, 1517 and 1333 mRNAs were predicted to be targets of 13-18 h, 18-24 h and 24-30 h DE miRNAs, respectively (Fig. 1B). These mRNAs were enriched in 7, 44 and 40 KEGG pathways, respectively. All the 7 pathways enriched by 13-18 h DE miRNAs were enriched as well by 18-24 h DE miRNAs, and 6 (except for the gap junction pathway) were enriched as well by 24-30 h DE miRNAs (Table 1). Among the 44 pathways enriched by the 18-24 h DE miRNAs, 38 were enriched as well by 24-30 h DE miRNAs. Thus, the number of the enriched pathways increased dramatically from 7 in 13-18 h oocytes to 44 in 18-24 h oocytes. Among the 6 pathways most enriched by 18-24 h DE miRNAs, 5 are most enriched as well by 24-30 h miRNAs (Table 1). The results confirm again that while miRNA profiles were similar between 13-h and 18-h oocytes and beween 24-h and 30-h oocytes, a marked change took place from 18 h to 24 h post hCG injection.

**Table 1:** Fold enrichment (FE) and P value of pathways enriched by mRNAs targeted by differentially-expressed (DE) miRNAs between 13 and 18 h, 18 and 24 h, and 24 and 30 h oocytes

KEGG Identifier	KEGG Pathway	13-18 h		18-24 h		24-30 h	
		FE	P	FE	P	FE	P
04920*	Adipocytokine SP	4.15(1)^*^	2.8E-03(1)	2.47(20)	5.3E-03(25)	2.58(8)	5.8E-03(16)
04540	Gap junction	3.64(2)	3.0E-03(2)	2.37(26)	2.5E-03(16)	-	-
05215	Prostate cancer	3.48(3)	4.0E-03(4)	2.26(29)	3.9E-03(23)	2.40(13)	3.3E-03(13)
04115	p53 SP	3.02(4)	4.7E-02(6)	**2.95(6)**	2.2E-04(11)	**3.33(3)**	5.6E-05(4)
05211	Renal C carcinoma	2.98(5)	4.9E-02(7)	**3.09(5)**	7.4E-05(7)	**3.08(5)**	2.5E-04(7)
04360	Axon guidance	2.39(6)	3.3E-02(5)	2.62(9)	7.3E-06(3)	2.52(9)	8.0E-05(5)
04010	MAPK SP	2.23(7)	3.5E-03(3)	2.21(31)	3.9E-07(2)	2.12(27)	1.1E-05(2)
00533	KS biosynthesis	-	-	**4.24(1)**	2.5E-02(42)	-	-
04130	SNARE interact	-	-	**4.02(2)**	1.1E-04(8)	**4.54(1)**	3.4E-05(3)
00601	Glycosphingolipid	-	-	**3.71(3)**	9.0E-03(30)	**3.60(2)**	2.2E-02(26)
04722	Neurotrophin SP	-	-	**3.43(4)**	1.5E-10(1)	**3.32(4)**	1.0E-08(1)
04340	Hedgehog SP	-	-	2.83(7)	2.7E-03(18)	2.40(15)	3.1E-02(33)
05220	Chronic ML	-	-	2.68(8)	6.8E-04(14)	2.46(12)	5.6E-03(15)
04912	GnRH SP	-	-	2.62(10)	1.5E-04(10)	2.22(23)	6.6E-03(17)
05223	Non-SCLC	-	-	2.59(11)	8.3E-03(28)	2.40(16)	3.1E-02(34)
04916	Melanogenesis	-	-	2.54(12)	2.3E-04(12)	2.16(25)	8.6E-03(19)
04720	L-term potentiation	-	-	2.54(13)	2.7E-03(19)	2.26(21)	2.2E-02(25)
04330	Notch SP	-	-	2.54(14)	1.4E-02(36)	**2.88(6)**	6.7E-03(18)
04910	Insulin SP	-	-	2.49(15)	1.9E-05(6)	2.19(24)	1.2E-03(10)
00510	N-Glyc biosynthesis	-	-	2.49(16)	2.5E-02(41)	2.50(11)	3.7E-02(38)
04310	Wnt SP	-	-	2.48(17)	9.7E-06(5)	2.03(29)	3.1E-03(12)
04664	Fc epsilon RI SP	-	-	2.48(18)	1.5E-03(15)	2.10(28)	2.5E-02(28)
05212	Pancreatic cancer	-	-	2.47(19)	3.5E-03(21)	2.40(14)	1.0E-02(20)
05014	ALS	-	-	2.46(21)	1.2E-02(33)	2.27(20)	4.1E-02(39)
05221	Acute ML	-	-	2.46(22)	1.2E-02(34)	2.52(10)	1.6E-02(22)
05213	Endometrial cancer	-	-	2.45(23)	1.8E-02(40)	-	-
05214	Glioma	-	-	2.39(24)	1.0E-02(31)	2.25(22)	3.1E-02(35)
04660	T cell receptor SP	-	-	2.37(25)	2.8E-04(13)	2.32(18)	1.2E-03(9)
05210	Colorectal cancer	-	-	2.37(27)	2.5E-03(17)	2.01(30)	3.4E-02(36)
04012	ErbB SP	-	-	2.34(28)	2.8E-03(20)	1.98(31)	3.7E-02(37)
04662	B cell receptor SP	-	-	2.23(30)	8.7E-03(29)	2.16(26)	2.1E-02(24)
04666	FcγR-m phagocytos	-	-	2.21(32)	3.7E-03(22)	2.35(17)	2.8E-03(11)
04350	TGF-beta SP	-	-	2.19(33)	7.2E-03(26)	2.64(7)	8.2E-04(8)
04520	Adherens junction	-	-	2.18(34)	1.4E-02(37)	-	-
04370	VEGF SP	-	-	2.18(35)	1.4E-02(38)	2.27(19)	1.5E-02(21)
04510	Focal adhesion	-	-	2.06(36)	1.3E-04(9)	1.82(34)	4.9E-03(14)
04120	Ubi-m proteolysis	-	-	1.96(37)	4.3E-03(24)	-	-
04914	P-m oo maturation	-	-	1.95(38)	3.2E-02(43)	-	-
05200	Pathways in cancer	-	-	1.93(39)	8.2E-06(4)	1.87(33)	9.1E-05(6)
04650	NK-m cytotoxicity	-	-	1.88(40)	1.4E-02(35)	1.77(37)	4.2E-02(40)
04114	Oocyte meiosis	-	-	1.88(41)	1.7E-02(39)	1.88(32)	2.7E-02(31)
04062	Chemokine SP	-	-	1.75(42)	7.7E-03(27)	1.66(38)	2.6E-02(29)
04810	R actin cytoskeleton	-	-	1.64(43)	1.1E-02(32)	1.59(40)	2.6E-02(30)
04144	Endocytosis	-	-	1.51(44)	4.3E-02(44)	1.64(39)	2.2E-02(27)
04630	Jak-STAT SP	-	-	-	-	1.80(35)	1.7E-02(23)
04110	Cell cycle	-	-	-	-	1.80(36)	3.1E-02(32)

“mmu” before numbers was omitted due to space limitation. ^**^ Numbers in brackets indicate ranking numbers of fold enrichment or P value at the same stage of oocyte aging. SP: signaling pathway; KS: keratan sulfate; SNARE interact: SNARE interactions in vesicular transport pathway; Glycosphingolipid: Glycosphingolipid biosynthesis; ML: myeloid leukemia; SCLC: small cell lung cancer; L-term: Long-term; N-Glyc: N-glycan biosynthesis ALS: Amyotrophic lateral sclerosis; FcγR -m: Fc gamma R-mediated phagocytosis; Ubi-m: Ubiquitin mediated proteolysis; P-m oo: Progesterone-mediated oocyte maturation; NK-m: Natural killer cell-mediated cytotoxicity; R actin cytoskeleton: Regulation of actin cytoskeleton.

### Functional verification of key proteins from the KEGG pathway enrichment analysis

When the biology of the predicted target genes was further explored regarding their molecular functions and involvement in the KEGG pathways, all the 7 KEGG pathways enriched by the 13-18 h DE miRNAs (Table 1) are related directly or indirectly to the MAPK pathway, which is involved in cell proliferation, differentiation and apoptosis [22]. Enrichment of the glycosphingolipid biosynthesis pathway suggests apoptosis, because glycosphingolipid has a role in apoptosis by hydrolyzing into pro-apoptotic ceramide [23,24]. Enrichment of the neurotrophin signaling pathway suggests involvement of brain-derived neurotrophic factor (BDNF), which has been found to promote oocyte maturation with antiapoptotic effects [25], and to function through engagement of BDNF receptor TrkB (tropomyosin receptor kinase B) [26].

**Figure 3:**
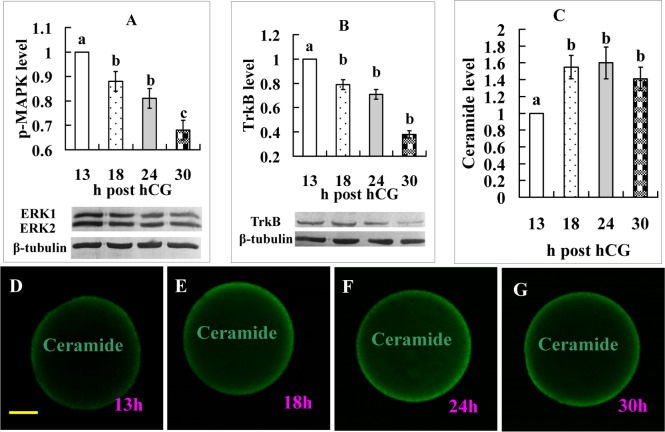
Levels of p-MAPK, TrkB, and ceramide expression in oocytes aging for different times Graphs **A**, **B** and** C** show quantification of p-MAPK, TrkB, and ceramide expression, respectively. For ceramide quantification, the fluorescence intensity value in oocytes recovered at 13 h post hCG injection was set as one and the other values were expressed relative to this value. Each treatment was repeated 3-4 times and each replicate contained 35-40 oocytes. a-c: Values with a different letter above bars differ significantly (P < 0.05). **D**, **E**, **F** and **G** are micrographs from laser confocal images showing expression of ceramide in oocytes recovered at different times after hCG injection. Ceramide was colored green in the laser confocal images. The bar is 18 μm and applies to all images.

**Figure 4:**
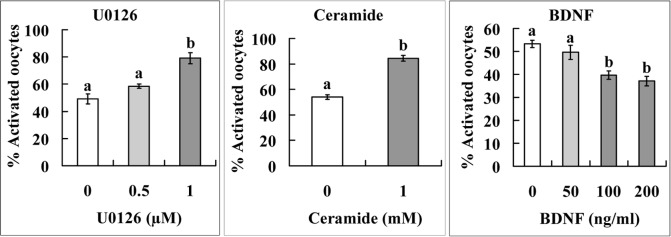
Effects of supplementation with U0126, ceramide or BDNF during in vitro aging on STAS of mouse oocytes Cumulus-denuded oocytes (DOs) recovered 13 h post hCG injection were used. For U0126 and BDNF treatment, the DOs were aged for 36 h in CZB medium supplemented with different concentrations of U0126 or BDNF before ethanol and 6-DMAP treatment for activation. For ceramide treatment, DOs were microinjected with 6 pl DMSO alone (control) or DMSO containing 1 mM ceramide and then aged for 36 h in CZB medium before ethanol and 6-DMAP treatment for activation. Each treatment was repeated 4-5 times with each replicate containing about 30 oocytes. a,b: Values with a different letter above bars differ significantly (P < 0.05).

Two experiments were performed to further verify functions of the above molecules during oocyte aging. In the first experiment, levels of p-MAPK, TrkB and ceramide expression were observed. The results showed that although the intra-oocyte levels of p-MAPK and TrkB decreased, the level of ceramide increased significantly with oocyte aging (Fig. 3). The second experiment determined the effects of supplementation with U0126 (a MAPK inhibitor), BDNF or ceramide during in vitro aging on oocyte susceptibility to activating stimuli (STAS). The results show that whereas U0126 and ceramide increased STAS, BDNF decreased STAS significantly (Fig. 4). The results further confirm that miRNAs play an important role in oocyte aging.

### MicroRNA-98 increases oocyte STAS

**Figure 5:**
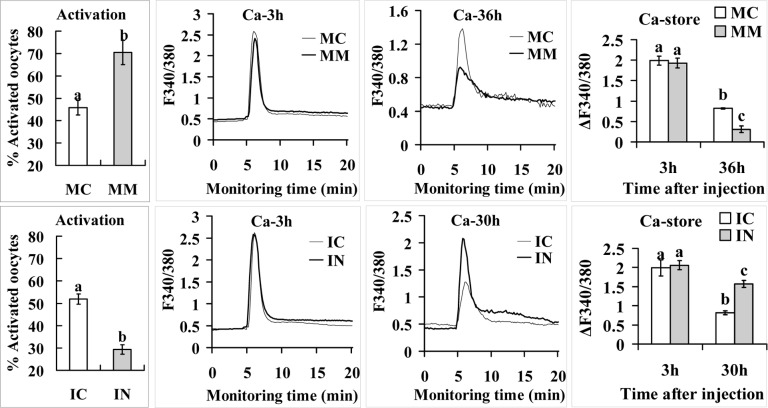
Activation rates and calcium stores in oocytes microinjected with miR-98 mimic (upper row) or inhibitor (lower row) Freshly ovulated oocytes recovered at 13 h post hCG were injected with miR-98 mimic (MM) or miRNA mimic negative control (MC), or with miR-98 inhibitor (IN) or inhibitor negative control (IC), before aging culture in CZB medium. To observe activation, oocytes were treated with ethanol and 6-DMAP at 36 h (mimic) or 30 h (inhibitor) of aging culture, and activation was checked 6 h later. Each treatment was repeated 3 to 4 times and each replicate contained 35-40 oocytes. For calcium measurement, at 3 h and 36 h (mimic) or 30 h (inhibitor) of aging culture, oocytes were loaded with Fura-2 AM and the loaded oocytes were measured for calcium stores (ΔF340/380) using a Ca^2+^ imaging system. Oocytes were monitored for 5 min to record baseline F340/380 ratio before ionomycin stimulation to release Ca^2+^ into cytoplasm. Following ionomycin addition, oocytes were monitored for 20 min to record peak F340/380 ratio. The difference between peak and baseline F340/380 ratios represents the calcium stores (ΔF340/380) of an oocyte. Each treatment was repeated 3 to 4 times and each replicate contained 35-40 oocytes. a-c: Values with a different letter above bars differ significantly (P < 0.05).

Mimics or inhibitors of miR-98 were microinjected into 13-h oocytes before aging culture. To observe STAS, injected oocytes were treated for activation with ethanol plus 6-DMAP at 36 h (mimic) or 30 h (inhibitor) of aging culture. Compared to control oocytes injected with mimic negative control (MC) or inhibitor negative control (IC), while oocytes injected with mimics (MM) showed significantly higher, those injected with inhibitors (IN) showed significantly lower rates of activation (Fig. 5). In other words, up- and down-regulating miR-98 expression promoted and alleviated oocyte aging, respectively.

### MicroRNA-98 impaired oocyte calcium stores while up regulating caspase-3

Because parthenogenetic activation of mammalian oocytes is associated with cytoplasmic Ca^2+^ increases [27,28], we observed effects of miR-98 on calcium stores of aging oocytes. Calcium stores in MC- or IC-injected control oocytes decreased significantly from 3 h to 30 h or 36 h after the injection (Fig. 5) suggesting that calcium stores in the endoplasmic reticulum decreased gradually with oocyte aging in vitro. By 36 h or 30 h of culture, however, while the calcium store in oocytes injected with miR-98 MM was lower, that in oocytes injected with IN was higher significantly than that in respective control oocytes. The results suggest that miR-98 increased oocyte STAS by facilitating calcium leak from the endoplasmic reticulum into the cytoplasm.

**Figure 6:**
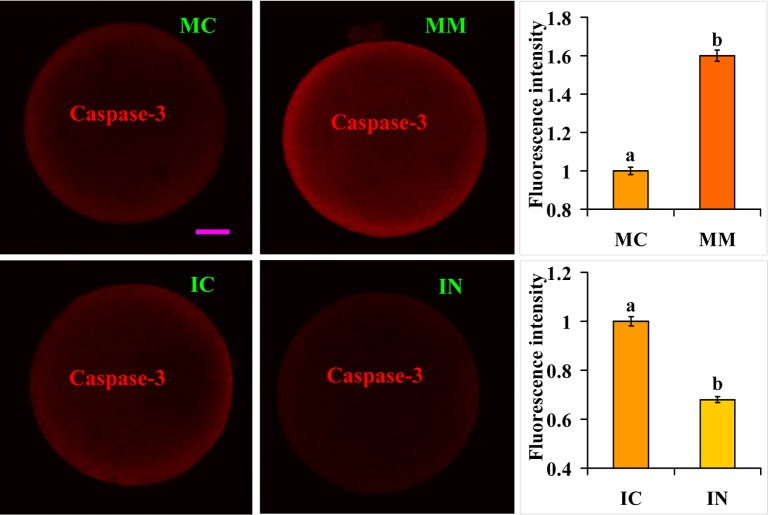
Caspase-3 expression in oocytes microinjected with miR-98 mimic or inhibitor Freshly ovulated oocytes recovered at 13 h post hCG were injected with miR-98 mimic (MM) or miRNA mimic negative control (MC), or with miR-98 inhibitor (IN) or inhibitor negative control (IC), before aging culture in CZB medium. At 36 h (mimic) or 30 h (inhibitor) of aging culture, oocytes were processed for immunofluorescence microscopy. Caspase-3 was colored red in the laser confocal images. The bar is 12 μm and applies to all images. For quantification, the fluorescence intensity values in control oocytes injected with MC or IC were set as one and the values in oocytes injected with MM or IN were expressed relative to the values in control oocytes. Each treatment was repeated 3-4 times and each replicate contained 35-40 oocytes. a,b: Values with a different letter above bars differ significantly (P < 0.05).

Target Scan Mouse (Release 6.2) analysis predicted that miR-98 might regulate caspase-3 expression. Furthermore, it has been reported that caspase-3 can cleave IP3 receptor and enhance calcium leak from the endoplasmic reticulum [29]. We thus hypothesized that miR-98 might facilitate calcium release by up regulating caspase-3 expression. The effect of miR-98 on caspase-3 expression was thus observed. At 36 h or 30 h of aging culture, caspase-3 expression was higher in oocytes injected with miR-98 mimics but was lower significantly in oocytes injected with inhibitors, compared with that in control oocytes injected with MC or IC (Fig. 6). The results suggest that miR-98 accelerated calcium release into cytoplasm by up regulating caspase-3 expression.

## DISCUSSION

Our results of hierarchical clustering and KEGG pathway enrichment analysis demonstrate that whereas miRNA expression in oocytes recovered at 13 h was similar to that in oocytes recovered at 18 h, a big change in miRNA expression profiles was observed from 18 to 24 h post hCG injection. Because our routine work shows that our mice ovulate about 12.5 h after hCG injection, oocytes recovered at 13, 18, 24 and 30 h post hCG injection would have aged in oviducts for 0.5, 5.5, 11.5 and 17.5 h after ovulation. Early studies showed that unfertilized oviductal mammalian eggs remained grossly unchanged for about 12 h after ovulation, and thus, morphological changes in mouse eggs due to aging became evident after they had spent approximately 12 h in the oviduct [30,31,32]. Whereas about 80% and 70% of the oocytes recovered at 12 h and 18 h post hCG injection developed into blastocysts, respectively, only 40% of the oocytes collected at 24 h after hCG injection completed blastocyst formation after fertilization in vitro [12]. Thus, the present results provide the first evidence that marked changes in miRNA expression are associated with significant alterations in function and morphology of aging oocytes.

Among the 6 apoptosis-related miRNAs we selected for RT-qPCR analysis, whereas miR-21 was antiapoptotic [33,34], miR-29b [35,36], miR-15a and miR-16 [37,38], miR-128 [39,40] and miR-98 [41,42; the present results] were pro-apoptotic. However, all these 6 apoptosis-related miRNAs significantly increased their expression from 18 to 24 h post hCG. While the increased expression of anti-apoptotic miR-21 can be considered as an effort of the aging oocyte make to fight against the growing apoptosis, the up regulation of the rest 5 pro-apoptotic miRNAs might have contributed to the apoptosis-related symptoms, notably the impaired developmental potential, during this period of oocyte aging. From 24 to 30 h post hCG, miRNAs tended to decrease their expression due possibly to oocyte deterioration. For example, our RT-qPCR showed that levels of all the analyzed miRNAs except for miR-128 decreased during this period. Furthermore, our microarray assay indicates that the overall number of down-regulated miRNAs remarkably increased with increasing time of oocyte aging.

An increase in miRNA expression was observed up to 24 h after hCG injection. It is known that oocytes accumulate a dowry of maternal mRNAs in preparation for embryogenesis [43]. These maternal transcripts are kept dormant and stored as mRNA ribonucleoproteins until late oogenesis or early embryogenesis when their translation is activated. Whether miRNAs produced during oogenesis are stored the same way is unknown. However, because the miRNA precursors (particularly the pri-miRNAs) share similarities in structure with mRNA precursors [44] and thus may be stored in the same way as RNA ribonucleoproteins, it is plausible that they were released from RNA ribonucleoproteins during oocyte aging and processed by Dicer into mature miRNAs, leading to the increased miRNA levels observed at 24 h post hCG. Expression of Dicer and miRNAs has been detected in metaphase II arrested oocytes [45,46]. As for the decrease in miRNA expression observed at 30 h, it might result from the exhaustion of the miRNA stores with oocyte degeneration, because oocytes at advanced stage of aging showed sever degenerative morphological changes [31], began cytoplasmic fragmentation (the current results) and lost the ability to be activated in vitro [30].

The early manifestations of aging oocytes include an increase in STAS [47,48], a decline in critical cell cycle factors such as MPF [30,49] and MAPK [49,50], and an impairment of developmental competence [12]. The late manifestations of aged oocytes include spindle abnormalities, losses of chromosomal integrity and cytoplasmic fragmentation [1]. Recent studies suggested that oxidative stress might act as the initiator for the aberrations observed in the aged oocyte. For example, oxidative stress can cause decreases in MPF and MAPK, impair calcium homoeostasis, induce mitochondrial dysfunction and directly damage multiple intracellular components of the oocyte such as lipids, proteins and DNA [1]. Zhang et al. [51] reported that SIRT1, 2, 3 protected mouse oocytes from post-ovulatory aging by suppressing oxidative stress.

Our functional analysis suggests that the KEGG pathways enriched by the 13-18 h DE miRNAs might be correlated with early manifestations of aging oocytes: all the pathways are involved directly or indirectly in regulating oxygen and calcium levels, MPF and MAPK activities, and/or cell apoptosis. The pathways most enriched by 18-24 h and 24-30 h DE miRNAs might be correlated with late symptoms of the aged oocytes. For example, the enrichment of the keratan sulfate (KS) biosynthesis pathway may be responsible for the decreased fertilizability of aged oocytes [12,30,49] as it may cause zona hardening and failure of sperm attachment. Several proteins of zona pellucida carry KS [52]. Cells do not bind lumican that carries sulfated KS chains [53]. Furthermore, KS reduces radiation-induced apoptosis of human cells [54]. The enrichment of the SNARE interactions in vesicular transport pathway might suggest increased vesicle fusion and oxidative stress. Premature exocytosis and inward migration of cortical granules have been observed in aged oocytes [12,49], which sometimes fused with lysosomes [55]. All the SNARE members for membrane fusion are present in porcine oocytes [56], and SNARE protein is up-regulated by oxidative stress in somatic cells [57]. The enrichment of the glycosphingolipid biosynthesis pathway suggests apoptosis, because glycosphingolipid facilitates apoptosis by hydrolyzing into ceramide [23,24]. Furthermore, in atretic oocytes, acid phosphatase positive membranes are organized in a whorl-like configuration reminiscent of the myelin body [58].

The current results show that miR-98 upregulated the expression of caspase-3, which enhanced the Ca^2+^ release from the endoplasmic reticulum, leading to increased STAS. Our recent studies demonstrated that oocyte increase in STAS was associated with cytoplasmic Ca^2+^ rises [28,59]. Although it has been observed that oocyte aging alters the regulation of the intracellular Ca^2+^ concentrations, affects Ca^2+^ oscillations and triggers apoptosis in fertilized eggs [60], the mechanisms underlying this defective Ca^2+^ release are not fully known. Increase in cytoplasmic Ca^2+^ concentration occurs by Ca^2+^ influx through the plasma membrane and by Ca^2+^ release from intracellular stores such as the endoplasmic reticulum [61]. The inositol 1,4,5-trisphosphate receptor (IP_3_R) is a tetrameric channel that accounts for a large part of the intracellular Ca^2+^ release. According to Nakayama et al. [62], the channel domain of IP_3_R1 tends to remain open and its large regulatory domain is necessary to keep the channel domain closed. It has been shown that caspase-3 cleaves IP_3_R1 during cell apoptosis [29,63] and oocyte aging [64,65], and the cleavage of IP_3_R1 may contribute to the cytosolic Ca^2+^ increase often observed in apoptotic cells and aging oocytes [66]. Thus, the present results provide evidence that miR-98 accelerated oocyte aging by promoting intracellular Ca^2+^ release via upregulating caspase-3.

Our result that miR-98 upregulated caspase-3 expression is in conflict with the general rule that miRNAs function by causing mRNA translational inhibition or degradation [20,21]. There are two possible mechanisms for miR-98 to upregulate caspase-3 expression. First, miR-98 upregulated caspase-3 expression indirectly by down regulating the expression of upstream inhibitors that would otherwise inhibit caspase-3 expression. Second, miR-98 directly upregulated caspase-3 expression. Emerging studies have revealed that miRNAs and their associated protein complexes (microRNPs) can additionally function to post-transcriptionally stimulate gene expression by direct and indirect mechanisms [67,68]. In addition, this study shows that miR-98 facilitated oocyte aging by increasing Ca^2+^ release through activating caspase-3. There are also reports that miR-98 promoted apoptosis by down regulating Bcl-xl [42], HMGA2 [41] or cyclin D2 [69]. However, other studies found that miR-98 suppressed apoptosis by down regulating Fas expression [70] or by directly targeting caspase-3 [71,72]. Thus, the mechanisms by which miR-98 regulates apoptosis are very complicated and need further investigations.

In summary, the current results showed that marked changes in miRNA expression were correlated with significant alterations in function and morphology of aging oocytes. These findings not only have confirmed critical roles for miRNAs in oocyte function, but also have opened up a new and interesting avenue towards an improved understanding of the underlying mechanisms for postovulatory oocyte aging. However, because this is the first study on the role of miRNAs in oocyte aging, for many issues, it is difficult to synthesize the current results to reach a definitive conclusion based on this single study. Further research is thus needed to verify the roles of miRNAs in oocyte function.

## MATERIALS AND METHODS

The chemicals and reagents used in this study were purchased from Sigma Chemical Co. unless otherwise specified.

### Oocyte recovery

Mice of the Kunming breed were kept in a room with 14L: 10D cycles, with the dark period starting from 20:00. The animals were handled according to the rules stipulated by the Animal Care and Use Committee of Shandong Agricultural University. Female mice, 8–10 weeks after birth, were induced to superovulate with 10 IU intraperitoneally (ip) equine chorionic gonado-trophin (eCG) followed by 10 IU ip human chorionic gonadotrophin (hCG) 48 h later. Both eCG and hCG used in this study were from Ningbo Hormone Product Co., Ltd. The superovulated mice were killed at different times after hCG injection, and the oviductal ampullae were broken in M2 medium to release oocytes.

### Oocyte activation

Our previous study showed that mouse oocytes were less susceptible to activating stimulus after aging in vitro than in vivo [30]. Our preliminary work showed that treatment with ethanol alone was a weak stimulus that activated only those oocytes that aged with MPF decreased to some extent, whereas ethanol treatment followed by post-culture with 6-dimethylaminopurine (6-DMAP) could activate oocytes less sensitive to activating stimulus without causing fragmentation. Thus, treatment with ethanol alone was used to evaluate activation susceptibility of in vivo aging oocytes, while ethanol plus 6-DMAP was used to evaluate the susceptibility of in vitro aging oocytes. Oocytes were first denuded of cumulus cells by pipetting in M2 medium containing 0.1% hyaluronidase. The cumulus-free oocytes were then treated with 5% (v/v) ethanol in M2 medium for 5 min at room temperature before culture in CZB medium alone (treatment with ethanol alone) or in CZB containing 2 mM 6-DMAP (ethanol plus 6-DMAP) for 6 h at 37°C in a humidified atmosphere of 5% CO_2_ in air. At the end of culture, oocytes were observed under a microscope for activation. Only those oocytes that had one or two pronuclei, or two cells each having a nucleus, were considered activated. Oocytes with more than two uneven cytoplasmic spheres were considered fragmented.

### Total RNA isolation

About 8 superovulated mice were sacrificed and about 240 oocytes were recovered at each time point after hCG injection on each experimental day. About 30 oocytes were randomly taken from the oocytes collected at each time point and subjected to ethanol alone activation to confirm the age of the oocytes. Only those oocytes whose activation rates were within the normal range observed in our routine work were used for RNA isolation. Whereas each treatment was repeated 5-6 times for microarray, each treatment was repeated 3 times for RT-qPCR assay. Thus, approximately 1200 and 600 oocytes were pooled to isolate RNA for microarray or qRT-PCR assay, respectively. Total RNA isolation from cumulus-free oocytes were performed using a mirVana™ miRNA Isolation Kit (Ambion, AM1560). The isolated RNA was then analyzed by Agilent 2100 to determine the integrity and concentration of total RNA.

### Expression profiling of miRNAs

The Agilent mouse miRNA microarrays (version 16.0, based on Sanger miRBase version 16.0) with a capacity to detect a total of 1023 miRNAs were used to compare the miRNA expression profiles of freshly ovulated oocytes and oocytes aged in vivo for various times. Cell lysate samples were submitted to Shanghai Biotechnology Corporation (Shanghai, China) for miRNA microarray assay. The miRNAs in total RNA were labeled using miRNA Complete Labeling and Hyb Kit (Cat#5190-0456, Agilent technologies, Santa Clara, CA, USA). One hundred nanogram Cy3-labeled RNA were hybridized to each microarray slide in hybridization Oven (Cat#G2545A, Agilent technologies, Santa Clara, CA, USA) at 55°C and 20 rpm for 20 h. After hybridization, slides were washed in staining dishes (Cat#121, Thermo Shandon, Waltham, MA, USA) with Gene Expression Wash Buffer Kit (Cat#5188-5327, Agilent technologies, Santa Clara, CA, USA). Slides were then scanned by Agilent Microarray Scanner (Cat#G2565BA, Agilent technologies, Santa Clara, CA, USA) and Feature Extraction software 10.7 (Agilent technologies, Santa Clara, CA, USA) with default settings. Raw data were normalized by Quantile algorithm, Gene Spring Software 11.0 (Agilent technologies, Santa Clara, CA, USA). The miRNAs with fold change greater than 2 (FC>2) were considered to be differentially expressed (DE) and subjected to further analysis. Target prediction of DE miRNAs was performed by four algorithms, DIANAmT, miRanda, PicTar4 and TargetScan. Only the mRNAs predicted by all the four algorithms were used to predict target mRNAs and the predicted target mRNAs were subjected to KEGG pathway analysis by DAVID [73].

### RT-qPCR analysis

To validate the microarray results, reverse transcription fluorescence quantitative real-time PCR (RT-qPCR) was conducted as described by Chen et al. [74] with modifications. Briefly, for miRNA stem-loop reverse transcription, 2 μl total RNA and 9 μl RNase free H_2_O were mixed and heated at 65°C for 10 min followed by quenching on ice. Then 4 μl 5× RT buffer, 0.5 μl RNase inhibitor (03531287001, Roche), 2 μl 10 mM dNTP (R0192, Fermentas), 2 μl 10 μM stem-loop reverse transcription primer (Table 2) and 0.5 μl reverse transcriptase (03531287001, Roche) were added and mixed to set up 20 μl reactions. The 20 μl reactions were incubated in Mastercycler (Eppendorf) for 30 min at 55°C, 5 min at 85°C and held at 4°C. Reactions for RT-qPCR including negative control were performed in triplicate. For RT-qPCR, the reaction mixture was prepared according to the manufacturer's instructions with Brilliant III Ultra-Fast QPCR Master Mix (600880, Agilent). The concentration of primers and Taqman probe were optimized and verified by standard curve. The concentration of forward and reverse primer was 600 nM and 300 nM, respectively. The concentration of Taqman probe was 200 nM for U6, mmu-miR-15a, mmu-miR-16, mmu-miR-21, mmu-miR-29b and mmu-miR-128, and 400 nM for mmu-miR-98. The sequences of primers and Taqman probe used were listed in Table 2. Reactions were run in Mx3005P (Stratagene) as follows: 95°C for 3 min followed by 40 cycles of 95°C for 20 s and 58°C for 20 s. The FAM and ROX fluorescence were collected after each cycle. Relative expression of miRNAs was calculated by MxPro software with 2-ΔΔCt and U6 as normalizer. The expression level of miRNAs in freshly ovulated oocytes was arbitrarily set as one.

**Table 2:** Sequences of stem-loop primers, RT-qPCR primers and Taqman probes used in this study

Gene	Accession	Primer Sequence
**Stem-loop primers**		
U6	NR_003027	5′-AACGCTTCACGAATTTGCGT-3′
mmu-miR-15a	MIMAT0000526	5′-CTCAACTGGTGTCGTGGAGTCGGCAATTCAGTTGAGCACAAACC-3′
mmu-miR-16	MIMAT0000527	5′-CTCAACTGGTGTCGTGGAGTCGGCAATTCAGTTGAGCGCCAATA-3′
mmu-miR-21	MIMAT0000530	5′-CTCAACTGGTGTCGTGGAGTCGGCAATTCAGTTGAGTCAACATC-3′
mmu-miR-29b	MIMAT0000127	5′-CTCAACTGGTGTCGTGGAGTCGGCAATTCAGTTGAGAACACTGA-3′
mmu-miR-98	MIMAT0000545	5′-CTCAACTGGTGTCGTGGAGTCGGCAATTCAGTTGAGAACAATAC-3′
mmu-miR-128	MIMAT0000140	5′-CTCAACTGGTGTCGTGGAGTCGGCAATTCAGTTGAGAAAGAGAC-3′
**RT-qPCR primers and Taqman probes**		
U6	NR_003027	F: 5′-CTCGCTTCGGCAGCACA-3′ R: 5′-AACGCTTCACGAATTTGCGT-3′ P: 5′-FAM-AGATTAGCATGGCCCCTGCGCAA-BHQ-3′
mmu-miR-15a	MIMAT0000526	F: 5′-ACACTCCAGCTGGGTAGCAGCACATAATGG-3′ P: 5′ FAM-TTCAGTTGAGCACAAACC-3′ BHQ
mmu-miR-16	MIMAT0000527	F: 5′-ACACTCCAGCTGGGTAGCAGCACGTAAATA-3′ P: 5′ FAM-TTCAGTTGAGCGCCAATA-3′ BHQ
mmu-miR-21	MIMAT0000530	F: 5′-ACACTCCAGCTGGGTAGCTTATCAGACTGA-3′ P: 5′ FAM-TTCAGTTGAGTCAACATC-3′ BHQ
mmu-miR-29b	MIMAT0000127	F: 5′-ACACTCCAGCTGGGTAGCACCATTTGAAATC-3′ P: 5′ FAM-TTCAGTTGAGAACACTGA-3′ BHQ
mmu-miR-98	MIMAT0000545	F: 5′-ACACTCCAGCTGGGTGAGGTAGTAAGTTGT-3′ P: 5′ FAM-TTCAGTTGAGAACAATAC-3′ BHQ
mmu-miR-128	MIMAT0000140	F: 5′-ACACTCCAGCTGGGTCACAGTGAACCGGT-3′ P: 5′ FAM-TTCAGTTGAGAAAGAGAC-3′ BHQ
URP		5′-TGGTGTCGTGGAGTCG-3′

F: forward; R: reverse; P: Taqman probe.

### Western blot analysis

Cumulus-free oocytes (n=350) were placed in a 1.5 ml microfuge tube containing 20 μl sample buffer (20 mM Hepes, 100 mM KCl, 5 mM MgCl_2_, 2 mM DTT, 0.3 mM phenylmethyl sulfonyl fluoride, 3 μg/ml leupetin, pH 7.5) and frozen at −80°C. For running the gel, 5μl of 5× SDS-PAGE loading buffer was added to each tube, and the tubes were heated to 100°C for 5 min. Total proteins were separated on a 12% polyacrylamide gel by SDS-PAGE and transferred electrophoretically onto PVDF membranes. After being washed in TBST (150 mM NaCl, 2 mM KCl, 25 mM Tris, 0.05% Tween 20, pH 7.4) and blocked with TBST containing 3% BSA for 1 h at 37°C, the membranes were incubated at 4°C overnight with rabbit anti-p-MAPK (Erk1/2) (Thr202/Tyr204) antibody (1:1000, 9101, Cell Signaling), or rabbit anti-TrkB (1:1000, ab18987, Abcam) polyclonal antibodies (1:1000, ab108319, Abcam) and mouse anti-β-tubulin monoclonal antibodies (1:1000, 05-661, Merck Millipore). Then, the membranes were washed in TBST and incubated for 1 h at 37°C with alkaline phosphatase-conjugated goat anti-rabbit IgG (1:1000, cw0111, Kangweishiji Biotechnology) or goat anti-mouse IgG (1:1000, cw0110, Kangweishiji Biotechnology). Finally, signals were detected by a BCIP/NBT alkaline phosphatase color development kit (Beyotime Institute of Biotechnology). The relative quantities of proteins were determined with Image J software by analyzing the sum density of each protein band image. The relative quantity values of p-MAPK and TrkB of 13-h freshly ovulated oocytes were set as one and the other values were expressed relative to this quantity.

### Immunofluorescence microscopy

Cumulus-free oocytes were fixed in 4% paraformaldehyde at 37°C for 15 min. Zona pellucida was removed by digestion in 0.5% pronase. Zona-free oocytes were permeabilized in 0.1% Triton X-100 at 37°C for 15 min and blocked for 30 min with 3% BSA. Oocytes were then incubated with mouse Anti-Ceramide antibody (1:50, C8104) or rabbit Active + Pro Caspase-3 antibody (100 fold diluted, ab47131, Abcam) at 37°C for 1 h followed by incubation at 37°C for 1 h with FITC-conjugated goat-anti-mouse IgG (400 fold diluted, 115-095-062, Jackson ImmunoResearch) or Cy3-conjugated goat-anti-rabbit IgG (200 fold diluted, 111-165-144, Jackson ImmunoResearch). Finally, the stained oocytes were mounted on glass slides and observed under a Leica laser scanning confocal microscope (TCS SP2). Argon (488 nm) and helium/neon (543 nm) lasers were used to excite FITC and Cy3, respectively. Fluorescence was detected with 505–540 nm (FITC) and 560–605 nm (Cy3) bandpass emission filters, and the captured signals were recorded as green and red, respectively. Caspase-3 and ceramide expression was quantified by analyzing fluorescence intensity using the ImagePro software.

### In vitro aging of oocytes

For in vitro aging, freshly ovulated cumulus-free oocytes were cultured (about 30 oocytes/100 μl drop) for different times in CZB medium supplemented with different concentrations of recombinant human BDNF or U0126 at 37°C in a humidified atmosphere of 5% CO_2_ in air. Recombinant human BDNF (450-02, PeproTech) and U0126 (U120) were dissolved in PBS and dimethyl sulfoxide (DMSO) at 100 μg/ml and 10 mM, respectively.

### Microinjection of miRNA mimics and inhibitors

Microinjection was performed under an inverted microscope equipped with differential interference contrast. Freshly ovulated cumulus-free oocytes were transferred into HCZB medium in a petri dish, covered with mineral oil and placed on the stage of the microscope. An oocyte was held to the holding pipette at the 9 o'clock position and then rotated until the oocyte side with the first polar body was around the 6 or 12 o'clock position. Five to 10 pl of 1 mM ceramide (43799) dissolved in DMSO or 50 μM mimics or 100 μM inhibitors or respective controls were injected into each oocyte using a micropipette with an inner diameter of 3 μm. The mimics, inhibitors and control injected included mmu-miR-98 mimics (miR10000545-1-2, Guangzhou RiboBio), miRNA mimic control (miR01101-1-2, Guangzhou RiboBio), mmu-miR-98 inhibitors (miR20000545-1-2, Guangzhou RiboBio) and miRNA inhibitor control (miR02101-1-2, Guangzhou RiboBio). Immediately after injection, oocytes were cultured (about 30 oocytes/100 μl drop) for aging in CZB medium at 37°C in a humidified atmosphere of 5% CO_2_ in air.

### Measurement for calcium stores

Cumulus-free oocytes were loaded with Ca^2+^ probe by incubating at room temperature for 30 min in HCZB medium containing 1 μM Fura-2 AM and 0.02% pluronic F-127. Oocytes were transferred into a HCZB medium drop in Fluoro dish (FD35-100, World Precision Instruments) covered with mineral oil and observed with Leica DMI6000 inverted microscope at 37°C. A Fura 2 fluorescence module was used for excitation, and a Leica LAS-AF calcium imaging module was used to calculate the F340/380 ratio, which represented the concentration of cytoplasmic calcium. The oocytes were monitored for 5 min to record the baseline F340/380 ratio before ionomycin stimulation to release Ca^2+^ into cytoplasm. For ionomycin stimulation, the drug was added to the HCZB medium drop to give a final concentration of 5 μM. Following ionomycin addition, the oocytes were monitored for 20 min to record the peak F340/380 ratio. The difference between the peak and baseline F340/380 ratios represented the calcium stores of an oocyte.

### Data analysis

At least three replicates were performed for each treatment. Percentage data were arc sine transformed and analyzed with ANOVA; the Duncan multiple comparison test was used to locate differences. The software used was SPSS (Statistics Package for Social Science). Data are expressed as mean ± SE and P < 0.05 was considered significant.
